# A Novel Oxygen Carrier (M101) Attenuates Ischemia-Reperfusion Injuries during Static Cold Storage in Steatotic Livers

**DOI:** 10.3390/ijms22168542

**Published:** 2021-08-09

**Authors:** Njikem Asong-Fontem, Arnau Panisello-Rosello, Alexandre Lopez, Katsunori Imai, Franck Zal, Eric Delpy, Joan Rosello-Catafau, René Adam

**Affiliations:** 1 Unité Chronothérapie, Cancers et Transplantation, Université Paris-Saclay, 94800 Villejuif, France; alexandregl.lopez@gmail.com (A.L.); rene.adam@aphp.fr (R.A.); 2 Experimental Hepatic Ischemia-Reperfusion Unit, Institut d’Investigacions Biomèdiques de Barcelona (IIBB), Spanish National Research Council (CSIC), 08036 Barcelona, Catalonia, Spain; arnau.panisello@iibb.csic.es (A.P.-R.); joan.rosello@iibb.csic.es (J.R.-C.); 3 Department of Gastroenterological Surgery, Graduate School of Life Sciences, Kumamoto University, Kumamoto 860-8555, Japan; katsuimai@hotmail.com; 4 Hémarina SA, Aéropôle Centre, 29600 Morlaix, France; franck.zal@hemarina.com (F.Z.); eric.delpy@hemarina.com (E.D.); 5 Centre Hépato-Biliaire, APHP Hôpital Universitaire Paul Brousse, Université Paris-Saclay, Villejuif, 94800 Paris, France

**Keywords:** ischemia-reperfusion, steatotic livers, oxygen-carrier

## Abstract

The combined impact of an increasing demand for liver transplantation and a growing incidence of nonalcoholic liver disease has provided the impetus for the development of innovative strategies to preserve steatotic livers. A natural oxygen carrier, HEMO2life^®^, which contains M101 that is extracted from a marine invertebrate, has been used for static cold storage (SCS) and has shown superior results in organ preservation. A total of 36 livers were procured from obese Zucker rats and randomly divided into three groups, i.e., control, SCS-24H and SCS-24H + M101 (M101 at 1 g/L), mimicking the gold standard of organ preservation. Ex situ machine perfusion for 2 h was used to evaluate the quality of the livers. Perfusates were sampled for functional assessment, biochemical analysis and subsequent biopsies were performed for assessment of ischemia-reperfusion markers. Transaminases, GDH and lactate levels at the end of reperfusion were significantly lower in the group preserved with M101 (*p* < 0.05). Protection from reactive oxygen species (low MDA and higher production of NO2-NO3) and less inflammation (HMGB1) were also observed in this group (*p* < 0.05). Bcl-1 and caspase-3 were higher in the SCS-24H group (*p* < 0.05) and presented more histological damage than those preserved with HEMO2life^®^. These data demonstrate, for the first time, that the addition of HEMO2life^®^ to the preservation solution significantly protects steatotic livers during SCS by decreasing reperfusion injury and improving graft function.

## 1. Introduction

Liver transplantation (LT) is the only treatment option that can potentially cure hepatocellular carcinoma and end-stage liver disease [1,2]. As the number of people diagnosed with liver disease increases, a growing number of patients are on waiting lists with a higher risk of mortality [3]. Nowadays, extended criteria donor (ECD) steatotic livers have become a solution to alleviate the shortage of organs. The obesity pandemic has resulted in the steatotic liver becoming the most common ECD graft [4]. It is well documented that when a graft macrosteatosis is greater than 30%, it increases the risk of a more severe reperfusion syndrome, primary non-function (PNF) and early allograft dysfunction (EAD), because of alterations in the microcirculation, extreme inflammatory response and mitochondrial impairment [5]; however, there are some cases in which well-selected patients have received a transplant using a 60% macrosteatosis graft with acceptable results [6,7].

The process of organ donation implies interrupting the blood circulation to the donor organ, leading to oxygen shortage, also known as ischemia, responsible for rapid cellular ATP decline, and the abrupt restoration of oxygen at reperfusion during liver transplantation, which is associated with detrimental oxidative stress with the potential to trigger inflammation. To mitigate this ischemia-reperfusion injury (IRI) phenomenon, it is a standard procedure to refrigerate the donor organ (cold storage), as a decrease in temperature causes a decline in ATP requirement slowing down the metabolism [8,9].

Another approach is to provide oxygen to the organ to increase the oxygen supply, preventing the sudden oxygen gap between ischemia and reperfusion. Sustaining ATP levels throughout the entire preservation steps prevents anaerobic metabolism and its detrimental byproducts, such as reactive oxygen species (ROS), which limits tissue injuries [10]. The most studied technic is HOPE (hypothermic oxygenated perfusion), in which oxygen is added directly to the preservation solution during dynamic preservation. Although this technic, first developed in the 1970s for kidneys [11], has shown its efficacy in multiple animal models and humans [12,13,14,15], it is not yet widely used in clinical practice due to cost and logistical constraints.

As a consequence, improving oxygen availability by adding an oxygen carrier (OC) directly to the preservation solution during cold storage is seen as an easier and cost-effective method to attenuate IRI. Several OCs were supplemented to preservation solution and their efficacy was compared [16]. Moreover, the addition of an OC was proposed to preserve cellular integrity and diminish the severity of the IRI phenomena [17,18,19,20].

Perfluorocarbons (PFCs) are high-capacity oxygen compounds with an oxygen solubility a hundred times higher than in blood. Supplementation of PFC in preservation solutions has been studied in a wide range of organs, including livers. Recent experimental studies on porcine models showed better preservation of aerobic metabolism leading to maintenance of mitochondria integrity and decrease inflammation [21]. However, not only a high partial O2 pressure is necessary to maximize O2 content, but it also equilibrates rapidly with the surrounding environment, quickly losing its oxygenation capacity [22].

As an alternative, M101 (HEMO2life^®^, Morlaix, France), an OC isolated from extracellular hemoglobin isolated from Arenicola marina, a marine invertebrate, reported encouraging results [23,24,25,26,27,28]. Indeed, unlike human hemoglobin that can carry 4 oxygen molecules, M101 can fix up to 156 molecules. M101 is not only active over a wide range of temperature (from 4 °C to 37 °C), but it also releases O2 through a gradient. This process contributes to continuous oxygen delivery and consumption by cells [29,30,31].

Here, we describe an experimental study using the Zucker rat lineage, an obese rat model of the steatotic liver where we studied the impact of M101 during static cold storage on the quality of organ preservation. For the first time, we compare conventional SCS of steatotic livers with a group of livers in which M101 is added to the preservation solution (PS). The hepatic function is analyzed for 2 h in an ex vivo perfusion system followed by histological and tissue analysis to decipher the impact of M101 on liver preservation.

## 2. Results

**Figure 1:**
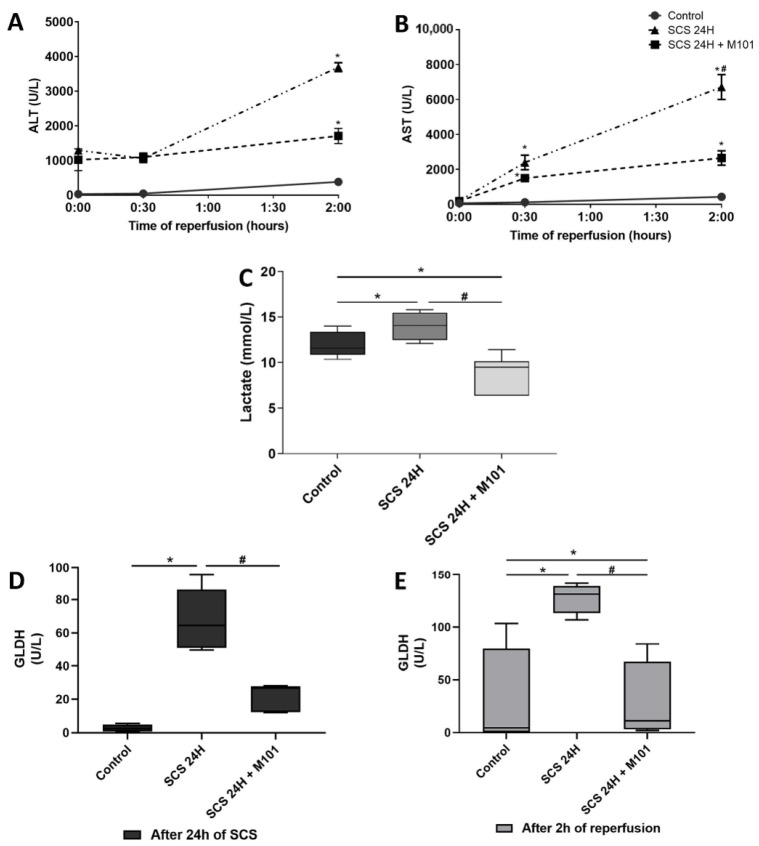
Hepatocellular and mitochondrial injury. (**A**) Aminotransferase (ALT) and (**B**) aspartate aminotransferase (AST) release remained lower in the control group than in both other groups during the 2 h of reperfusion. At the end of the reperfusion, transaminases release in the SCS-24H + M101 group was decreased as compared with SCS-24H group. Data are represented as mean (SEM). (**C**) Lactate levels were significantly lower in the SCS-24H + M101 group than in the control and SCS-24H groups after 2 h of reperfusion. The control group lactate levels were decreased, as compared with the SCS-24H group. (**D**,**E**) Glutamate dehydrogenase (GLDH) expression after 24 h of SCS was higher in the SCS-24H group. Following the 2 h of reperfusion, the level of GLDH in the SCS-24H group remained significantly the highest. Data are represented as mean (SD). *n =* 12/group, * = *p* < 0.05 vs. control and # = *p* < 0.05 vs. SCS-24H (Mann–Whitney–Wilcoxon).

In our model, liver function was assessed after two hours of normothermic machine perfusion (NMP) to mimic the physiological conditions found after liver transplantation is performed. The hepatocellular injury was evaluated through the quantification of the transaminases and lactate levels during the NMP. In addition, GLDH was quantified both at the end of the cold storage and after NMP. After two hours of reperfusion, the levels of AST and ALT were significantly lower in the group preserved with M101 (SCS-24H + M101) as compared with the SCS-24H group. The anaerobic state of the hepatocyte was reflected by the lactate levels, that were significantly decreased in the SCS-24H + M101 group, as compared with the SCS-24H group. No statistical significance was observed between the control group and the group preserved with M101. As expected, the control group had a very low amount of GLDH. Indeed, GLDH was increased in the SCS-24H group and significantly decreased in the preserved livers of the SCS-24H + M101 group. Similar results were obtained after reperfusion (Figure 1).

**Figure 2:**
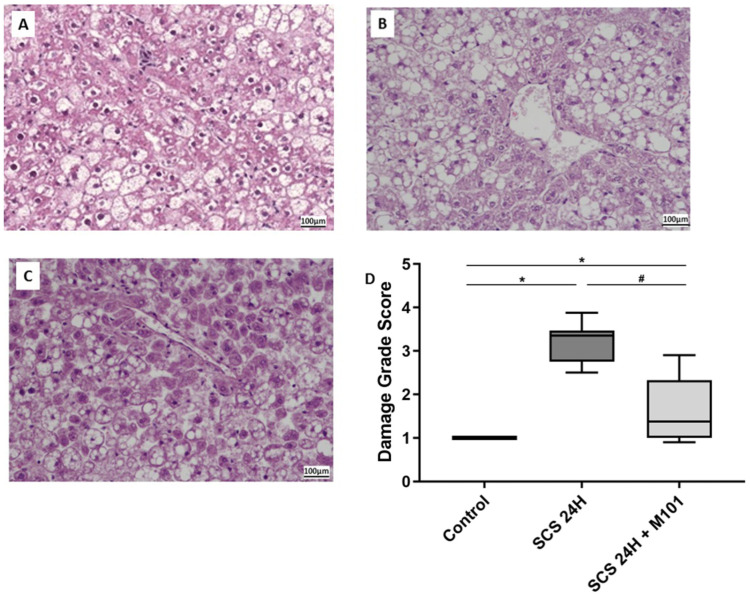
Histological damages reduction with M101. Histology of (**A**) control group; (**B**) SCS-24H group; (**C**) SCS-24H + M101 group. The damage grade score (**D**) is based on necrosis and coagulation, acute necrosis, disorganization, granular precipitate, sinusoidal dilatation, eosinophilic deposits, number of cells remaining in vessels and inflammation, observed by an expert pathologist. Data are represented as mean (SD). *n =* 12/group, * = *p* < 0.05 vs. control and # = *p* < 0.05 vs. SCS-24H (nonparametric one-way ANOVA (Kruskal–Wallis test)).

The histological damage score analyses showed from moderate (34–66%) to severe (>66%) infiltration of macro- and micro-vesicular steatosis in all groups (Grade II–III). The control group showed a preserved hepatic architecture. Similarly, the SCS-24H + M101 group presented a well-preserved hepatic anatomy showing low sinusoidal dilatation and cell swelling. On the contrary, the SCS-24H group presented fragmentation of the hepatic structure and more severe cellular damage. Indeed, the liver cellular dissociation and swelling were exacerbated, as compared with the SCS-24H+ M101 group (Figure 2) (SCS-24H group vs. SCS-24H + M101: 3.198 vs. 1.724, *p* < 0.05).

**Figure 3:**
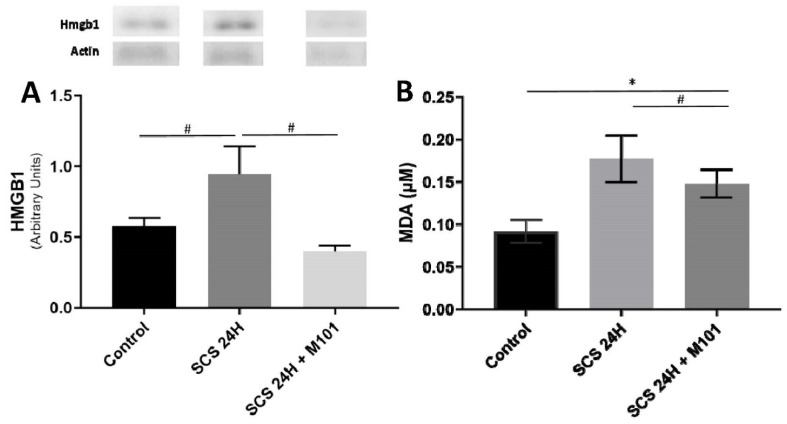
Nuclear injury and oxidative stress. (**A**) HMGB1 expression. (**B**) Malondialdehyde (MDA) levels were both significantly higher in livers preserved 24 h in SCS without the addition of M101, than without SCS (control) or with supplementation of M101. Data are represented as mean (SEM). *n* = 12/group, * = *p* < 0.05 vs. control and # = *p* < 0.05 vs. SCS-24H (one-way ANOVA).

Free radicals and inflammatory mediators play a key role in liver transplantation (LT) outcome because of their deleterious effects on the cell membrane and subsequent tissue damage after IRI [32,33]. It is well known that MDA also inflicts organelle damage at different structural levels and signaling pathways, exposing the cell to a high level of stress and, finally, death. MDA was significantly higher in the SCS-24H group and this was correlated with a higher quantification of High Mobility Group Box 1 (HMGB1), a transcription factor reflecting DNA breakage when released in the cytoplasm (Figure 3). Both parameters were significantly decreased by the presence of M101 in the preservation solution.

**Figure 4:**
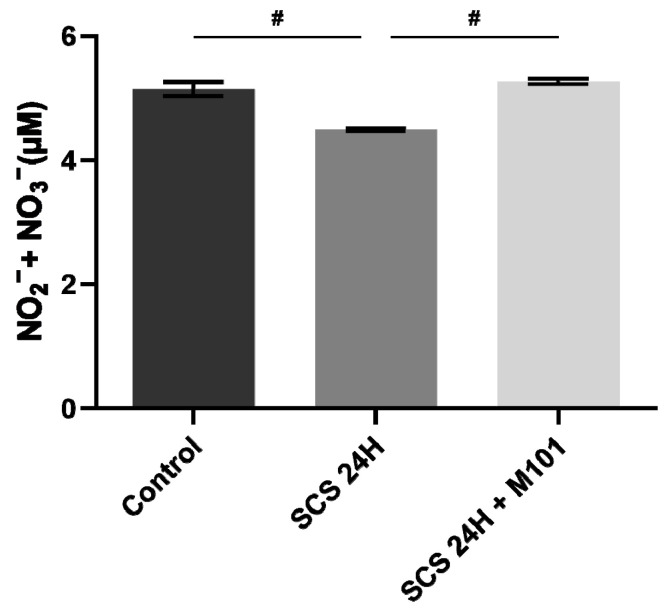
Nitrate/nitrite production decreased in the absence of M101. Nitrate and nitrite production was significantly decreased in the SCS-24H group after the 2 h reperfusion. Data are represented as mean (SEM). *n* = 12/group, # = *p* < 0.05 vs. SCS-24H (Mann–Whitney–Wilcoxon).

As a decreased nitrous oxide (NO) expression is observed in human patients showing liver ischemia reperfusion injuries [34], we measured nitrate plus nitrite compounds, which are indicators of NO production. The levels of nitrite and nitrate were decreased with the PS alone and returned to the control levels when the PS was supplemented with M101 (Figure 4).

**Figure 5:**
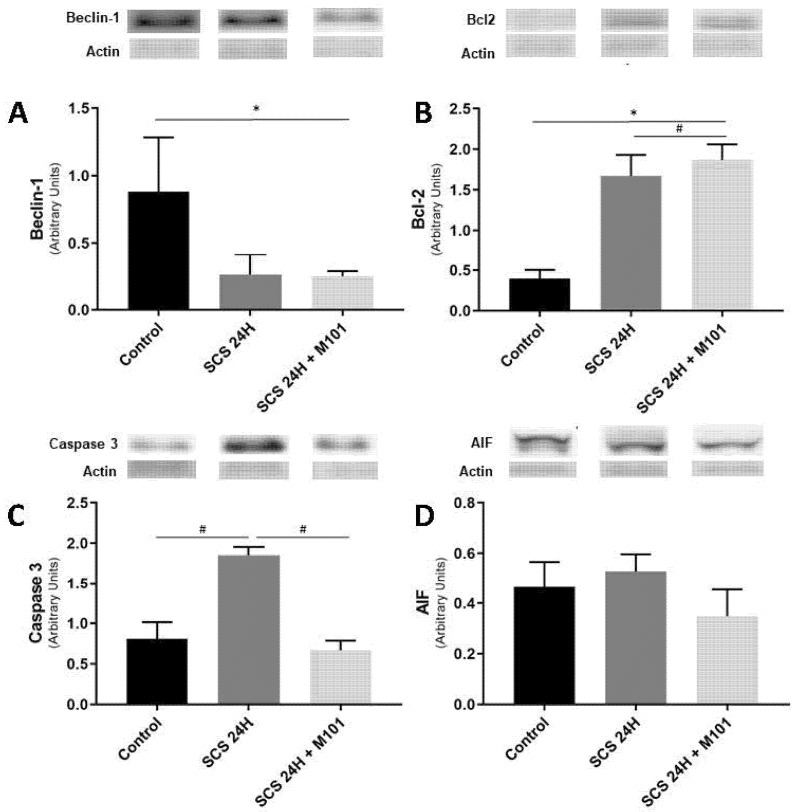
M101 impact on autophagy and apoptosis related marker. (**A**,**B**) Beclin-1 significant downregulation and Bcl-2 significant upregulation in the SCS-24H + M101 group seems to be in favor of the activation of autophagy. (**C**,**D**) Apoptosis effectors tend to be downregulated in the SCS-24H + M101 group, as compared with the control group and with the SCS-24H group. Data are represented as mean (SEM). *n* = 12/group, * = *p* < 0.05 vs. control and # = *p* < 0.05 vs. SCS-24H (one-way ANOVA).

It is well known that cell degradation is regulated by the interaction of Beclin-1 and Bcl-2 and Bcl-2 can inhibit apoptosis and autophagy [35,36]. Beclin-1 expression was the lowest and Bcl-2 the highest in the SCS-24H + M101 group, as compared with the SCS-24H group and the control group. Caspases are proteases that also play a major role in apoptosis and in necrosis and inflammation [37]; therefore, we quantify caspase-3, an executioner caspase which is activated once cleaved. Caspase-3 expression was significantly higher in the SCS-24H group than in the group preserved with M101 and the control group that showed similar levels. We did not find any significant differences between the SCS-24H + M101 group and the SCS-24H group for the measurement of the expression of the apoptosis-inducing factor (AIF), when compared to the control group (Figure 5). However, both apoptosis pathway markers tend to be downregulated in the SCS-24H + M101 group, suggesting a protection against ischemia reperfusion injuries.

## 3. Discussion

At the present time, steatotic livers from ECDs are more commonly offered to transplantation centers. New strategies to protect these fragile grafts from IRI are being developed, such as NMP, to evaluate their functions in real-time before transplantation, or defatting strategies, that have been described as potential tools to augment the donor pool. The problem is that it requires technical knowledge which increases the final cost of the procedure [5,38,39]; however, every single organ is submitted to a period of static cold storage (SCS) and preservation solution (PS) additives seem to overcome most of the obstacles seen in NMP or defatting.

M101 is a promising additive to PS especially in the context of transplantation because of its exceptional capabilities to carry O2 molecules, but also its non-immunogenicity and its absence of toxicity. Moreover, it has been demonstrated that M101 supplementation led to better long-term outcomes in pig kidneys [40] and non-steatotic livers [26], both in static and dynamic preservation. In this study, we demonstrated the capacity of supplementing the PS with M101 to protect steatotic livers during SCS, as compared with the standard technique. Transaminases are the most common markers used to measure the integrity of the hepatocytes. We showed that transaminases were lower in livers stored with M101. Failures in liver transplantation were linked to elevated lactate levels observed at the end of the graft preservation period [41,42,43]. In our study, we observed lower lactate levels in the group preserved with M101 than SCS alone for 24 h. This is consistent with the work of Le Pape et al., who demonstrated that adding M101 in media was associated with lower production of LDH [44]. Therefore, the addition of an M101 oxygen carrier in PS seems to enhance the capability of the graft to tolerate the ischemic period by lowering anaerobic metabolism detrimental by-products.

These results were consistent with greater prevention of mitochondrial damage, as determined by the GDH activity levels in livers preserved with M101, as compared with livers preserved in SCS alone. Oxidative stress triggers the opening of pores, altering the permeability of the mitochondria. When this membrane collapses, there is a reduction in ATP production and cytochrome C is released and, subsequently, fatally damages the cell [45]. Moreover, the addition of M101 also maintains mitochondrial complex 1 activity, allowing oxidative phosphorylation to be preserved during cold ischemia [31]. Therefore, M101 seems to have a positive effect on the maintenance of mitochondrial activity, which is key to improve limit oxidative stress caused by ROS.

Indeed, ROS are generated mainly in the mitochondria (especially during reperfusion), leading to the generation of lipoperoxides. MDA is a product of lipid peroxidation of mitochondrial membranes and may be cytotoxic [46,47]. The fact that MDA levels were lower with M101 could enable the graft to better perform after reperfusion.

Recently, many studies have shown that NO plays a versatile mediating role in multiple signaling pathways, such as the inhibition of platelet aggregation, regulating microcirculation [48] and preventing apoptosis downregulation caspases activities [49,50,51,52]. Indeed, NO inhibits caspase-3 proteases via S-nitrosylation, preventing their activations and inhibiting apoptosis [53]. In this study, we show that NO tends to be higher in the M101 group, which is protective, allowing the liver to cope better with ischemia-reperfusion injuries.

In the context of IRI, autophagy of dysfunctional mitochondria prevents the release of cytochrome c, which can have a protective or an injurious role during IRI [54], depending on the phase. Indeed, during cold and warm ischemia, its expression is protective, as it regulates ATP consumption through the recycling of amino acids and prevents ROS formation, delaying apoptosis. However, during reperfusion, its long-term expression could lead to the engulfment of essential organelles, leading to hepatocyte death. Key markers of autophagy are BECN-1 and BCL-2. An intricate interplay exists between autophagy and apoptosis as both are involved in similar signaling pathways. Indeed, when BCL-2 interacts with BECN-1, it inhibits autophagy and the apoptotic pathway may be favoured [55,56]. On the other side, Bcl-2 interaction with Back/Bax prevents apoptosis. In our study, low Beclin 1 expression coupled to high Bcl-2 expression in the SCS 24h + M101 group vs. control suggests that M101 could protect steatotic livers through the autophagy pathway, which is protective during cold ischemia.

Nuclear injury caused by apoptotic cells is characterized by DNA cleavage, freeing DNA bound transcriptions factors, such as the oxidative stress sensor Hmgb1 [57]. Indeed, its extracellular location is recognized as damage-associated molecular pattern (DAMPs) by the immune system and activates inflammation. Hmgb1 accumulation leads to a competitive exchange with Bcl-2. Hmgb1 interaction with Bcl-1 promotes the autophagosome formation, which is the initiation stage of autophagy [58]. Here, we demonstrated that M101 lowers Hmgb1 expression, protecting steatotic livers from inflammation and activation of the intrinsic apoptotic signaling pathway.

Altogether, NO, Bcl2, Beclin-1 and Hmgb1 levels suggest that M101 seems to protect steatotic liver preservation by favouring autophagy over apoptosis. These conclusions are also supported by AST, ALT and lactate expression. Indeed, as their expression peak reflects the level of necrosis and apoptosis of hepatocytes [26], we showed that M101 supplementation significantly decreases the peak. Low level of AIF and total caspase-3 expression also partially confirms our analysis, though caspase-3 activity needs to be investigated in further studies. All these results are consistent with histological findings.

M101 presents many advantages, compared to other oxygen carriers (OCs). One characteristic of M101 is its compatibility in all preservation solutions. This advantage is demonstrated in preventing IRI in thoracic and abdominal organs [23,24,25]. The quality of the grafts was far superior compared to the SCS alone, regardless of the PS used. These previous studies are concordant with our present research. Other similar approaches have been scrutinized, such as the hemoglobin-based OCs; however, these are NO scavengers and, unfortunately, lead to vasoconstriction and hypertension [59,60]. M101 is also superior to other OC because of its passive release of O2 in an oxygen gradient-dependent manner. Indeed, it does not require an allosteric effector, when the right oxygen amount is in the environment [61]. M101 is also one of the only OC compatible for a wide range of temperatures, meaning that it can be used in hypothermia as well as normothermia. Altogether, M101 physico-chemical features make it one of the most investigated OCs for organ transplantation. As it has already shown its safety and efficacy in preventing IRI in humans’ kidneys and heart preservation [23,24,27,29], further investigations need to be made on livers, more specifically, to determine the oxygen quantity which needs to be added to the preservation solution to optimize M101 function.

Our study was performed on the Zucker rat lineage, a model of choice to study the specificity of steatotic liver preservation. Although these grafts are known to be more sensitive to IRI, their use has great potential for increasing the pool of donors. Therefore, developing innovative and easy-to-implement strategies to lower their sensitivity towards IRI is critical in liver transplantation.

With this aim, our study investigated the benefit of adding an OC to the PS and provided preliminary evidence for a new strategy to prevent IRI in ECD grafts. Indeed, we prove, for the first time, that the addition of M101 to the preservation solution used for steatotic livers seemed to attenuate anaerobic metabolism, autophagy and apoptosis.

Our results should encourage further investigations using other animal models and other protocols, such as dynamic preservation strategies (e.g., HOPE and NMP), to deepen the understanding of how oxygen carriers such as M101, found in HEMO2life^®^ or other additives, can help mitigate IRI.

## 4. Materials and Methods

### 4.1. Animals

Homozygous obese (OB) male Zucker (Crl: ZUC(Orl)Leprfa) rats, aged 12–14 weeks, weighing 505 g (+/−48 g) were purchased (Charles River, Italy). The rats were housed in separate cages with a closed ventilation system, 2 rats per cage, in a room with controlled humidity and temperature and a 12 h light cycle. Water and food were provided ad libitum. Rats were chosen randomly and did not receive food or water for 20 h before the experiment. This study adhered to European Union regulations (EC-guideline 86/609/CEE) and was approved by the Ethics Committees for Animal Experimentation (CEEA) of the University of Barcelona (number 483116; approved on 14 July 2016).

### 4.2. HEMO2life^®^ and Preservation Solution

The oxygen carrier (HEMO2Life^®^, Hemarina, Morlaix, France) was stored at −80 °C and each vial was thawed individually to 4 °C and added to the PS to achieve a final concentration of 1 g/L, according to the manufacturer’s procedures. The PS (IGL-1^®^, Lissieu, France) was selected because it is the most commonly used PS in France and has shown advantages in liver transplantation [62].

### 4.3. Liver Procurement and Experimental Groups

**Figure 6:**
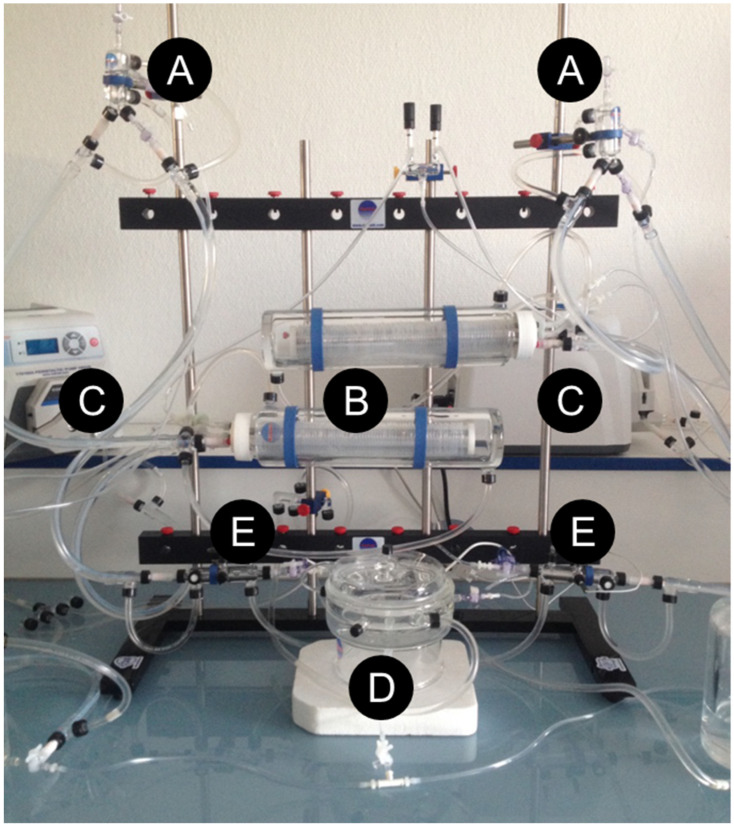
Experimental ex vivo machine perfusion system. Ex vivo machine perfusion system is adapted from liver/kidney systems. (**A**) Bubble trap. (**B**) Membrane oxygenating. (**D**) Liver perfusion chamber (Radnoti LLC, Covina, CA, USA), assembled by the authors (N.A.-F. and A.L.). (**C**) Peristaltic pump (MasterFlex *^®^*, Thermo Fisher Scientific, Waltham, MA, USA). (**E**) Flow sensor (TS410 Tubing Module, Transonic Systems Inc., Ithaca, NY, USA), a water bath circulator pump (9102A12E, 6 L High-Stability Digital Controller Refrigerated/Heated Circulating Bath, Polyscience, Niles, IL, USA) and an oxygen bottle (95% O2/5% CO_2_, Air Liquide, Paris, France).

Liver procurement was performed in a sterile room, under general anesthesia through a silicon mask, at 2 L/min airflow and 4% isoflurane for induction, followed by 2% for maintenance. Through a midline incision, the gastrointestinal tract was covered with a wet gauze and placed to the left. The hepatic ligaments were cut on the left section of the diaphragm and then the right suprarenal vein was closed with 8–0 non-absorbable polypropylene sutures. Heparin (0.3 UI/gr) was injected intravenously. After clamping the supra celiac abdominal aorta before its iliac bifurcation, the aortic cannulation with a 25-gauge catheter was immediately rinsed with 50 mL of PS (IGL-1^®^, Lissieu, France), using a syringe pump (Pilote C^®^, Fresenius, France). The liver was flushed with 20 mL of PS through a fixed 21-gauge catheter in the portal vein; then, the graft was submerged in a sterile bath at 4 °C, after dividing the supra and infra hepatic inferior vena cava and the rest of the hepatic hilum. Evaluation of the liver functions after SCS was performed using a normothermic machine perfusion (NMP) ex vivo system (Figure 6).

**Figure 7:**
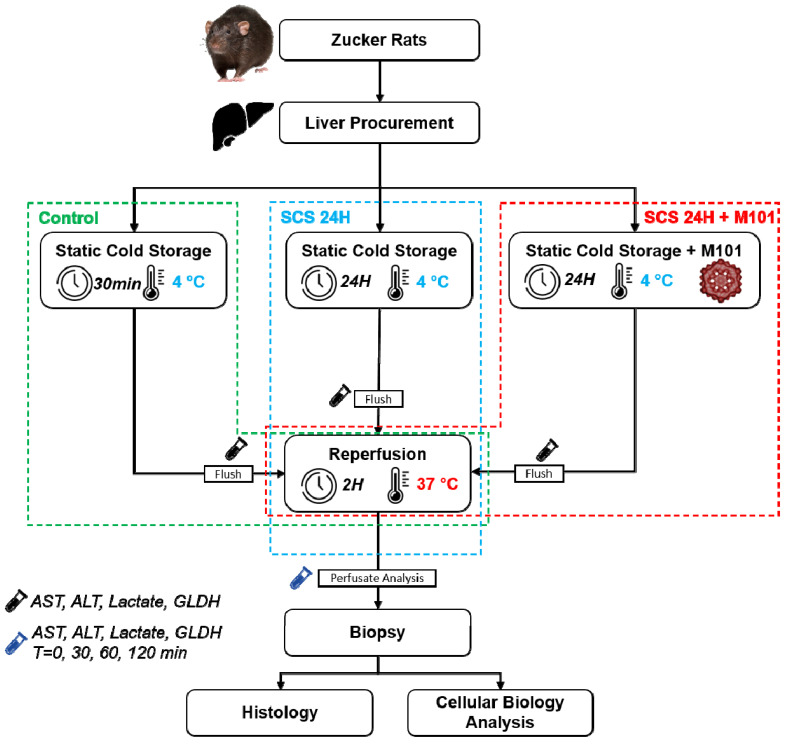
Schema experimental protocol. Rat livers were directly subjected to 30 min of static cold storage (control) followed by 2 h of normothermic reperfusion. Livers were rinsed with IGL-1 preservation solution supplemented (static cold storage (SCS-24H + M101 group) or not (SCS-24H group) with 1 g/L of M101, followed by 24 h of SCS and 2 h of reperfusion. At the end of the reperfusion, biopsies were taken and directly put into nitrogen (−80 °C) for cellular biology analysis (Western blot) or in PFA for histology. Perfusates were collected starting from the flushing (0 min) and at different time points during reperfusion (30 min, 1 h and 2 h).

A total of 36 animals were randomly assigned to the following 3 experimental groups: (1) control group (*n =* 12 livers), immediately perfused in the ex vivo system at 37 °C for direct evaluation; (2) SCS-24H group (*n =* 12 livers), preserved for 24 h in preservation solution (PS) under static cold storage (SCS) alone before 2 h of normothermia machine perfusion (NMP) evaluation at 37 °C, as the current gold standard; (3) SCS-24H + M101 group (*n =* 12), where the PS was supplemented with M101 and the livers were preserved and evaluated using the same procedure as the former group (Figure 2). Perfusate and tissue samples were taken during and after the experiments, frozen in liquid nitrogen and kept at −80 °C for further analysis (Figure 7).

### 4.4. Histology

After two hours of reperfusion at 37 °C in the ex vivo system, the liver tissues were fixed in 10% paraformaldehyde, embedded in paraffin and cut in 3 µm sections, before being stained with hematoxylin–eosin–safran (HES), according to standard procedures. The damage grade score is based on necrosis and coagulation, acute necrosis, disorganization, granular precipitate, sinusoidal dilatation, eosinophilic deposits, number of cells remaining in vessels and inflammation. Each component was carefully evaluated by an expert pathologist and assigned a value from 1 to 4 (1, absence; 2, low presence; 3, mild presence; 4, high presence).

### 4.5. Biochemical Determination

The perfusate analysis of lactate, alanine aminotransferase (ALT) and aspartate aminotransferase (AST) was conducted in the clinical laboratory at our institution. Glutamate dehydrogenase (GDH) (GL441, Randox Lab, Crumling, UK) was measured by quantifying the decline in absorbance at 340 nm, according to the manufacturer’s protocol.

### 4.6. Western Blot

Liver tissue was homogenized in HEPES buffer and proteins were separated by SDS-PAGE and transferred to PVDF membranes using the iBlot Dry Blotting System (Invitrogen AB, Lidingö, Sweden). Membranes were immunoblotted using the following antibodies: HMGB1 (ab79823, Abcam, Cambridge, UK), caspase-3 (#9662, Cell Signaling Technology, MA, US), apoptosis-inducing factor (AIF) (ab32516, Abcam, Cambridge, UK), Beclin-1 (ab207612, Abcam, Cambridge, UK) and B-cell lymphoma 2 (Bcl-2) (ab136285, Abcam, Cambridge, UK). After washing, the bound antibody was detected after incubation for 1 h, at room temperature, with the corresponding secondary antibody linked to the horseradish peroxidase enzyme. Quantification was performed using ImageJ (Rasband, W.S., ImageJ, U. S. National Institutes of Health, Bethesda, MD, USA, https://imagej.nih.gov/ij/, 1997–2018, Accessed date 30 July 2021).

### 4.7. ELISA

Quantification of malondialdehyde (MDA) (ab118970, Abcam, Cambridge, UK) and nitrite/nitrate NO (Item No. 780001, Cayman Chemical, Ann Arbor, MI, USA) in tissue samples were obtained according to the manufacturer’s instructions. Optical density was quantified using a Modulus II microplate reader (Turner Biosystems, Sunnyvale, CA, USA).

### 4.8. Statistics

Data from the damage grade scores were compared statistically by using a nonparametric one-way ANOVA test (Kruskal–Wallis test) with Dunn’s post-hoc analysis. All data are expressed as means (±SD or ±SEM). Data were compared statistically using a one-way ANOVA test with a post-hoc Tukey’s test for multiple comparisons (GraphPad Prism version 8.0.0 for Windows, GraphPad Software, San Diego, CA, USA, www.graphpad.com, Accessed date 30 July 2021). *p* < 0.05 was considered to be significant.

## Data Availability

The data presented in this study are available on request from the corresponding author.
